# Elemental redistribution in tropical soils: insights into REE, U, and Th mobility after extended phosphogypsum use

**DOI:** 10.1007/s10653-026-02991-6

**Published:** 2026-02-07

**Authors:** Luís Paulo P. Tanure, Isabela C. F. Vasques, Renato W. Veloso, Maria Maiara C. Tanure, Walter A. P. Abrahão, Carlos Roberto Bellato, Massimo Gasparon, Jaime W. V. de Mello

**Affiliations:** 1 Departamento de Solos, Universidade Federal de Viçosa, Avenida Peter Henry Rolfs S/N, Campus Universitário, Viçosa, MG CEP 36570-900 Brazil; 2 Instituto Federal de Educação, Ciência e Tecnologia de Goiás, IFG, 21 St., Area Especial 4, Águas Lindas de Goiás, GO Brazil; 3 Instituto Federal de Educação, Ciência e Tecnologia de Mato Grosso, IFMT, Alta Floresta Rodovia MT 208, S/N - Lote 143-A, Loteamento Aquarela - Hamoa, Cuiabá, MT Brazil; 4 Departamento de Química, Universidade Federal de Viçosa, Campus Universitário, Viçosa, MG Brazil; 5 EIT RawMaterials, Knesebeckstraße 62-63, 10719 Berlin, Germany; 6 School of the Environment, The University of Queensland, St Lucia, QLD-4072 Australia; 7 INCT Acqua, Universidade Federal de Minas Gerais, Av. Antônio Carlos, 6627, Belo Horizonte, MG CEP 31270-901 Brazil

**Keywords:** Actinides, Lanthanides, Trace elements

## Abstract

Phosphogypsum is a soil conditioner used to enhance lime effects in depth and also to decrease aluminum (Al) toxicity to plants. It is a by-product from phosphate mining and can be enriched in some contaminants, such as rare earth elements (REE), uranium (U), and thorium (Th). However, there is a lack of studies evaluating the long-term effects of this practice, particularly in soil depths. To address this gap, this study aimed to evaluate the remobilization of REE, U, and Th in a highly weathered soil (Oxisol) for 9 years. Four soil parcels received a single application of 28 tons ha^−1^ in different years: 2005, 2008, 2010, and 2013, with soil sampling occurring in 2014. Soil samples were digested and analyzed using mass spectrometry. The REE input from phosphogypsum reached 1681 mg kg^−1^, of which 95.89% corresponded to light rare earth elements (LREE). Although the reference area showed high background levels, temporal enrichment occurred in the treated areas, especially for LREE in the 0–40 cm layer, with cerium (Ce) being the most abundant element. Over nine years, REE concentrations decreased, but the distribution patterns of REE, U, and Th revealed to be dependent on chemical characteristics, natural background in soil and content in the phosphogypsum. The ratio ΣLREE/ΣHREE normalized by the reference area increased over the years, meaning a preferential depletion of HREE. Over time, phosphogypsum inputs led to enrichment of Th and scandium (Sc), whereas U and Y showed higher mobility and losses. These findings highlight the temporal and spatial behavior of potentially toxic elements introduced via phosphogypsum and their implications for soil and environmental quality.

**Graphical abstract:**

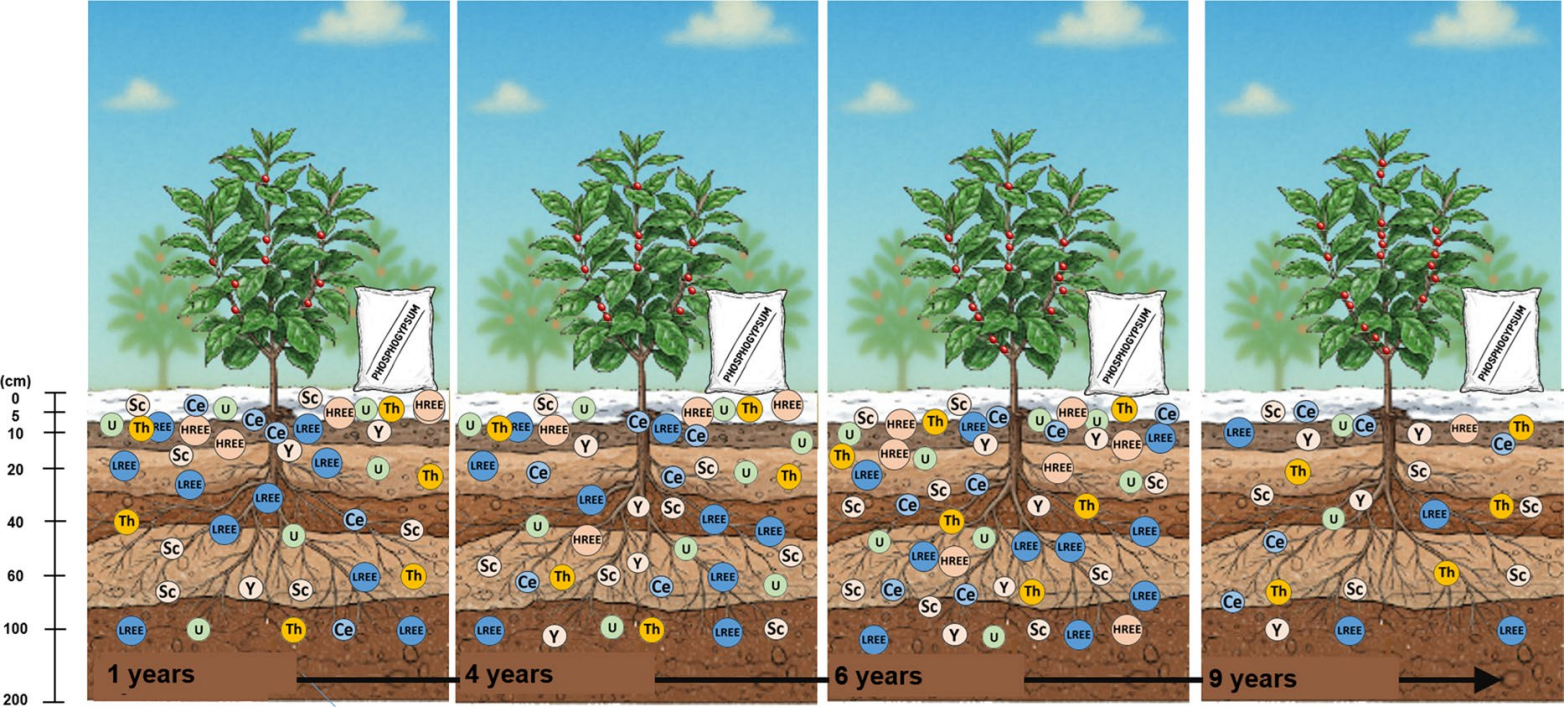

**Supplementary Information:**

The online version contains supplementary material available at 10.1007/s10653-026-02991-6.

## Introduction

In many developed and developing countries, soils are affected by waste disposal and also by the use of by-products from mining and industry. Such procedures potentially contribute to the introduction of trace elements into the soils. Among these trace elements are the rare earth elements (REE), uranium (U), and thorium (Th) (Luo et al., 2024). Likewise other potentially toxic elements, their presence in the environment can cause adverse effects (Zhang et al., 2025; Ferreira et al. 2022).

The REE correspond to the lanthanides (Ln) from lanthanum (La, Z = 57) to lutetium (Lu, Z = 71), including yttrium (Y, Z = 39) and scandium (Sc, Z = 21) (IUPAC, 1990; Chakhmouradian & Wall, 2012). U (Z = 92) and Th (Z = 90), in turn, belong to the actinide series and are typically found in minerals associated with REE. In soils, REE have been discussed in several studies with different focuses, from review articles dealing with overall contents of REE in specific soils (Mihajlovic & Rinklebe, 2018) to ecological risks of their exposure (Zhang et al., 2024, 2025).

According to the atomic number, REE are typically divided into two groups: light rare earth elements (LREE), a group formed from La to Gadolinium (Gd), and heavy rare earth elements (HREE), which include elements from Terbium (Tb) to Lu, along with Y and Sc. Y is considered a HREE due to its ionic radius (0.9 Å) being similar to that of Holmium (Ho) (Castor & Hedrick, 2006). The similarity between their physicochemical properties explains their widespread occurrence in the lithosphere and also their behavior in the environment (Hu et al., 2006; Tyler, 2004a).

Approximately 200 distinct mineral species containing REE have already been described (Kanazawa & Kamitani, 2006). In Brazil, these elements are found in different minerals, with the main sources being monazite, pyrochlore, barite, magnetite, and apatite (Biondi and Braga Júnior, 2023). Apatite is the main mineral found in phosphate deposits in Brazil, and the city of Araxá produces approximately 20,000 tons/year of ground apatite for use as a phosphate source in agriculture (Bonotto, 2020). The processing of this mineral in the fertilizer industry aims to obtain phosphoric acid and generates agricultural gypsum as a byproduct, also referred to as phosphogypsum, primarily composed of calcium sulfate (CaSO_4_) and water.

The phosphogypsum is very soluble in soils, which promotes calcium (Ca) movement to greater depths, reduces aluminum (Al) saturation in the subsurface, and stimulates root development at depth (da Costa & Crusciol, 2016). As a result, it favors the absorption of water and nutrients from deeper layers during dry periods. Phosphogypsum is also used in saline soils to correct sodium or potassium saturation (Abril et al., 2008). Therefore, it is an important amendment for minimizing the effects of soil acidity in the subsurface, complementing lime application by promoting deeper increases in soil hydrogen ion potential (pH) and improving Ca^+2^ supply (Bossolani et al., 2023). However, since REE concentrations in phosphate fertilizers have raised concerns (Brilhante et al., 2025), similar attention has been directed toward REE levels in phosphogypsum (Ramos et al., 2016a).

The presence of REE in phosphogypsum has already been reported in many studies (El Zrelli et al., 2019; Qing et al., 2025) and is attributed to the presence of REE minerals and isomorphic substitution of Ca^2+^. The growing interest in REE and the principles of the circular economy highlight the critical need to understand the fate of these elements when introduced into the environment through materials such as phosphogypsum (Akfas et al., 2024; Ramos et al., 2016b; Wu et al., 2018). However, many studies have aimed to increase the efficiency of REE recovery from phosphogypsum (Gasser et al., 2022), and few studies have explored the behavior and mobility of REE, U, and Th in soils (Rout et al., 2022). Notably, there is a lack of investigations addressing the long-term mobility of REE, U, and Th in highly weathered tropical soils under field conditions (Kang et al., 2024).

Similarly, REE, U, and Th, when present in agricultural inputs and/or in the soil, can exhibit analogous behavior to calcium, particularly La (Ramos et al., 2016b). Therefore, these elements may display mobility in the soil profile, depending on their intrinsic characteristics, and distinct mobility patterns can be observed between LREE and HREE. However, other variables may affect their behavior, including the amount of phosphogypsum applied, the soil’s cation exchange capacity (CEC), adsorption by clay and oxides (Zhang et al., 2024), the complexation of sulfates into soluble compounds (da Costa & Crusciol, 2016), the pH of the solid matrix (Kachoueiyan et al., 2024), and the ionic strength of the system (Bishop et al., 2024).

To address the knowledge gap regarding phosphogypsum application and mobilization of REE, U, and Th, this study aims to evaluate their concentration along the soil profile. Tropical soils of widespread occurrence were examined to determine the duration of phosphogypsum application influence in total chemical concentrations. This study brings the importance of field-based investigations to assess the vertical distribution of these elements and compare them with a reference soil from a native forest area. It is expected that this study will provide a clearer understanding of the dynamics of these critical elements in the environment and support decision-makers in establishing guidelines for phosphogypsum application.

## Material and methods

### Sampling area

Sampling was conducted in coffee farms in São Roque de Minas and Piumhi, State of Minas Gerais, Brazil, at the coordinates 20°15′S and 46°18′W; 20°11′S and 46°22′W; 20°23′S and 45°59′W. These coordinates refer to the farms and not to the soil profiles. The geology of the region is characterized by the Sete Lagoas Formation, which belongs to the Paraopeba Subgroup of the Bambuí Group and consists of sedimentary rocks such as limestone, dolomite, and metapelitic rocks (CPRM, 2003).

The sampling areas feature a rolling terrain with convex slopes and convex or tabular summits, with slopes ranging from 5 to 24°. These areas are in the physiographic region of Alto São Francisco, in the central-western part of the state of Minas Gerais (Figure S1). The climate of the region is classified as Cwa according to the Köppen classification, with a well-defined dry season from May to September, an average annual precipitation of 1,300 mm, and an average temperature of 20.7 °C.

The area was established with coffee cultivation (*Coffea arabica* L.) in a *Latossolo Vermelho* classified according to the Brazilian Classification System (SBCS, 2018) or Oxisol, according to the Soil Taxonomy (Soil Survey Staff, 2014). The soil samples were collected in April of 2014, and the four areas had received phosphogypsum in 2013, 2010, 2008, and 2005, that is, sampling occurred 1, 4, 6, and 9 years after application, respectively. All areas received a single application of 28 t ha^−1^ (7 kg m^−1^) of Araxá phosphogypsum in the planting row at the time of crop establishment.

For cultivation, the soil was prepared by subsoiling to 0.06 m, followed by the use of a cultivator equipped with a rotary hoe that loosened a 0.5-m-wide strip to a depth of 0.6 m. This process allowed the mixing and incorporation of fertilizers into the planting row, as recommended for the crop. Immediately after transplanting the seedlings, phosphogypsum was applied to the soil surface along the planting row, covering a 0.3-m-wide strip.

The samples were collected by opening a trench, and the center of the soil profile was defined as the sampling area. The sampling sequence started from the deepest to the shallowest layer to avoid contamination. The experimental design consisted of four replicates, in which the times (years passed after the phosphogypsum application: 1, 4, 6, and 9) characterized the main plots, and the sampled depths were considered subplots. Each plot was sampled at seven depths (cm): 0–5; 5–10; 10–20; 20–40; 40–60; 60–100; and 100–200. In total, 112 soil samples were collected: seven depths, four areas, and four replicates per site.

### Chemical, physical, and mineralogical analysis

The 112 samples were air-dried, sieved through a 2-mm mesh, homogenized, and quartered. Chemical and physical analyses (Tables 1 and 2) were performed according to Jackson (2005) and Teixeira et al., (2017). The 2-mm fraction was chosen because it corresponds to the air-dried fine earth (ADFE), which includes only the sand, silt, and clay fractions of the soil and is commonly used for chemical and physical analyses (Teixeira et al., 2017). For the determination of weathering indices Ki and Kr, sulfuric acid digestion was performed based on the procedure defined by Teixeira et al., (2017). Ki and Kr were calculated as molecular ratios, being Ki: 1.7 × SiO_2_ /Al_2_O_3_ and Kr [1.7 × SiO_2_/(Al_2_O_3_ + (0.64 × Fe_2_O_3_))].

**Table 1:** Chemical characterization of the profile of four sampled areas collected in April 2014, which had received phosphogypsum in 2013, 2010, 2008, and 2005

Depth (cm)	pH	P	S	K ^+^	Ca^2+^	Mg^2+^	Al^3+^	H ^+^ Al	t	T	V	m	OM
		mg dm^−3^			cmol_c_ dm^−3^						%		dag kg^−1^
0 to 5	4.81	71.70	277.10	116.67	8.69	0.53	0.55	9.43	10.58	18.95	41.63	12.37	6.42
5 to 10	5.57	9.70	19.77	111.67	11.93	0.33	0.33	7.80	4.93	12.43	36.97	8.83	5.81
10 to 20	5.53	4.47	14.10	96.33	3.38	0.17	0.32	8.20	4.12	12.00	31.23	12.07	5.47
20 to 40	5.01	4.23	24.77	63.33	1.75	0.07	0.98	9.03	2.81	11.02	17.70	37.10	5.17
40 to 60	4.87	0.50	37.90	40.00	1.56	0.04	0.75	9.07	2.45	10.77	15.80	30.73	4.56
60 to 100	4.85	0.23	67.70	27.00	1.71	0.04	0.81	8.27	2.63	10.39	18.00	30.93	4.18
100 to 200	4.91	0.20	68.97	13.67	2.61	0.07	0.65	7.87	3.37	10.58	25.47	19.63	3.62
2008													
0 to 5	4.65	1.20	17.53	363.33	2.31	1.26	0.46	5.83	13.13	10.33	45.07	10.87	5.17
5 to 10	5.13	32.67	536.90	229.33	55.38	0.89	0.39	4.83	20.33	25.21	75.37	2.27	4.22
10 to 20	5.04	33.73	573.37	123.00	15.05	0.51	0.16	4.97	16.04	20.84	67.57	1.60	4.31
20 to 40	5.12	1.23	115.00	35.33	4.21	0.34	0.17	4.43	4.60	9.07	51.03	3.57	3.44
40 to 60	5.32	0.10	94.27	17.33	3.46	0.36	0.10	3.23	3.96	7.10	54.47	2.17	2.20
60 to 100	5.75	0.03	71.83	10.67	3.30	0.37	0.00	2.37	3.70	6.37	61.03	0.00	1.55
100 to 200	5.48	0.10	27.47	11.67	2.24	0.49	0.00	1.80	2.76	4.56	59.93	0.00	1.03
2010													
0 to 5	4.75	17.40	354.10	168.33	14.47	0.27	0.62	6.43	5.13	21.60	51.53	17.83	5.21
5 to 10	5.08	3.00	101.87	210.33	10.97	0.26	0.39	5.73	4.85	9.92	45.13	9.77	4.44
10 to 20	5.23	3.07	88.23	201.00	3.61	0.24	0.16	4.67	4.52	9.03	47.77	4.73	3.96
20 to 40	5.14	1.10	82.37	87.67	2.65	0.15	0.20	4.30	3.31	7.32	40.93	9.10	3.32
40 to 60	5.47	0.53	78.57	17.33	3.24	0.24	0.13	3.53	3.66	7.06	49.60	5.23	2.76
60 to 100	5.82	0.00	50.20	8.67	3.20	0.34	0.00	2.20	3.57	6.06	62.03	0.00	1.72
100 to 200	5.30	0.00	19.97	10.33	0.95	0.28	0.00	2.20	1.26	3.46	34.33	0.00	1.16
2013													
0 to 5	5.95	349.90	916.10	278.00	54.02	1.13	0.13	4.07	36.84	59.93	93.83	0.17	5.51
5 to 10	5.50	76.10	562.40	1065.33	46.08	1.21	0.33	5.27	19.63	24.84	69.17	4.23	4.44
10 to 20	5.59	32.33	245.03	817.33	5.25	1.62	0.13	4.47	9.09	13.43	65.57	2.50	4.39
20 to 40	5.31	1.17	23.10	362.67	3.28	1.05	0.23	4.47	4.70	9.72	53.40	7.23	3.45
40 to 60	5.19	0.27	26.73	118.33	1.86	0.60	0.20	4.33	2.96	7.10	40.00	8.87	2.75
60 to 100	5.10	0.73	19.30	96.00	1.43	0.47	0.13	4.63	2.27	6.67	31.77	5.23	2.28
100 to 200	4.69	0.77	15.67	70.00	0.58	0.24	0.13	4.73	1.13	5.73	17.13	12.00	1.85

**Table 2:** Soil texture analysis and sulfuric digestion for two depths of the studied soil

Depth (cm)	Sand	Silt	Clay	^*^SiO_2_	Al_2_O_3_	Fe_2_O_3_	Ki	Kr
	g kg^−1^							
10–20	50	180	770	102	151.58	89.65	1.14	0.83
100–200	50	140	810	105	158.36	94.21	1.12	0.81

^*^Data obtained from the work of Ramos et al., 2013

Chemical and physical characterizations were carried out without replicates but in a certified laboratory. Physical characterization was performed for only one soil profile, as the soils were assumed to differ solely in their chemical composition.

The pH was determined in water using a soil:solution 1:2.5. Available phosphorus and potassium were extracted by Mehlich extraction (HCl 0,05 mol L^−1^ + H_2_SO_4_ 0.0125 mol L^−1^), using a 1:10 soil:solution ratio. Sulphur was extracted using Ca(H_2_PO_4_)_2·_H_2_O in HOAc 2 mol L^−1^. Available Ca, magnesium (Mg) and Al were extracted with KCl 1 mol L^−1^. Potential acidity (H + Al) was extracted by Buffer SMP (from Shoemaker, McLean, Pratt) (Shoemaker et al., 1961). CEC was determined as the sum of Ca, Mg, Al, Na, and Al (t). The potential CEC also considers the H ^+^ activity (T). The base saturation was calculated as the CEC occupied by Ca, Mg, Na, and Al while Al saturation was calculated as the proportion of Al in the CEC. Organic matter was determined by oxidation with K_2_Cr_2_O_7_ 2 mol L^−1^ + H_2_SO_4_ 5 mol L^−1^. All analyses were conducted according to Teixeira et al., (2017).

Undisturbed samples were also collected using volumetric rings from the A and Bw horizons of the trenches to determine hydraulic conductivity as well as the macro and microporosity of the soil (Table S1). The determination of particle density, soil bulk density, total pore volume, hydraulic conductivity, and grain size analysis were performed according to the methodology recommended by Teixeira et al., (2017).

For the mineralogical analyses, two samples from the 10–20 cm and 100–200 cm depths of a representative profile were used; that is, the same soil profile selected for physical characterization (Table 2). To obtain the clay fraction of the samples, dispersion was performed with 0.1 mol L^−1^ NaOH and a Na_2_CO_3_ solution at pH 9.5 (Jackson, 2005). Oriented slides were prepared by the smear method, using the clay in its natural and de-ironized state. After air-drying, the slides were analyzed using X-ray diffraction on a PANalytical diffractometer, model X'Pert Pro, with a cobalt anode tube, and the power unit operated at 40 kV and 40 mA. The diffractograms (Fig. S2) obtained were interpreted based on the Selected Powder Diffraction Data for Minerals.

### Determination of REE, U, and Th

After air-drying, the samples were quartered to obtain subsamples of 5 g each. The subsamples were ground in an agate mortar and sieved through a 200 mesh (0.074 mm) sieve. All the material was transferred to pre-cleaned polyethylene tubes.

The samples were dried at 40 °C for 24 h in a stainless-steel oven with forced air circulation. Subsequently, the EPA 3051A method from the Environmental Protection Agency (U.S.EPA, 2007) was performed, using 9.0 mL of nitric acid and 3.0 mL of hydrochloric acid, both double-distilled and P.A. from Merck. The extracts were filtered, and the concentrations of REE, U, and Th were determined by Inductively Coupled Plasma Mass Spectrometry (ICP-MS) (Perkin Elmer equipment, model NexIon 300D). The acid digestion was performed in triplicates.

All measurements were performed under previously optimized conditions, as follows: isotopes were selected based on their natural abundance and the likelihood of spectral interferences. An internal standard (Rhodium - Rh 5 μg L^−1^) was used to correct interferences. The eleven-point calibration curves were prepared for each element analyzed from a standard solution of 10,000 mg L^−1^. Re-calibration was performed when the standard deviation between blank measurements or checkpoint (2.0 µg L^−1^) measurements were > 10%. All elements presented calibration curves with correlation coefficients (r) better than 0.998. A certified reference material (Montana Soil II) was used to ensure quality. The detection limit is provided in the supplementary material (Table S2). The values obtained, with their respective standard deviations, were as follows (in mg kg^−1^): Sc = 4.14 ± 0.16; Y = 15.71 ± 0.60; La = 24.74 ± 2.13; Ce = 46.29 ± 3.63; Pr = 6.47 ± 0.37; Nd = 22.55 ± 1.59; Sm = 4.69 ± 0.25; Eu = 1.46 ± 0.06; Gd = 5.50 ± 0.35; Tb = 1.78 ± 0.03; Dy = 4.79 ± 0.26; Ho = 1.73 ± 0.05; Er = 3.34 ± 0.05; Tm = 2.74 ± 0.02; Yb = 3.36 ± 0.27; Th = 4.68 ± 0.43 and U = 1.46 ± 0.08. These results attest to the accuracy of the analytical process.

## Reference area and phosphogypsum

To compare the results regarding the time effect, soil samples from an area of native forest near the sampling sites were analyzed following the same sampling procedures and used as reference samples (Table 3). The contribution of phosphogypsum from Araxá (extracted in Brazil, city of Araxá) to the addition of REE, U, and Th in the soil was also evaluated. To estimate the input of phosphogypsum in the surface soil layer (0–5 cm), the amount applied, the application range (30 cm), the thickness of the surface layer (5 cm), and the soil bulk density were considered, all within 1 m of the planting row. No temporal analysis was performed for the reference area because all sampling occurred in 2014.

**Table 3:** Average concentration of the elements of interest in the samples of Oxisol from the reference area (without phosphogypsum application)

Depth (cm)	LREE, U and Th								
	La	Ce	Pr	Nd	Sm	Eu	Gd	Th	U
	mg kg^−1^								
0–5	1.99	111.31	0.65	2.54	0.69	0.15	2.10	7.02	2.57
5–10	2.18	112.64	0.71	2.60	0.69	0.14	2.05	6.82	2.49
10–20	2.10	109.06	0.65	2.42	0.69	0.14	2.03	6.96	2.54
20–40	2.15	113.30	0.68	2.19	0.71	0.16	2.13	6.89	2.49
40–60	2.29	115.71	0.73	2.65	0.71	0.16	2.23	7.16	2.55
60–100	2.41	128.47	0.75	2.77	0.77	0.17	2.39	7.34	2.72
100–200	2.05	113.12	0.65	2.45	0.70	0.15	2.10	6.78	2.53

	HREE								
Depth (cm)	Sc	Y	Tb	Dy	Ho	Er	Tm	Yb	Lu
	mg kg^−1^								
0–5	14.94	1.52	0.10	0.51	0.08	0.24	0.03	0.19	0.03
5–10	14.79	1.52	0.10	0.48	0.08	0.22	0.03	0.20	0.03
10–20	14.69	1.50	0.10	0.49	0.08	0.23	0.03	0.20	0.02
20–40	13.64	1.58	0.10	0.51	0.09	0.23	0.03	0.20	0.03
40–60	14.68	1.66	0.11	0.51	0.09	0.25	0.03	0.21	0.03
60–100	15.55	1.74	0.12	0.57	0.10	0.27	0.04	0.23	0.03
100–200	13.84	1.60	0.10	0.51	0.08	0.23	0.03	0.21	0.02

### Statistical analysis

The results obtained from ICP-MS for REE, U, and Th were submitted to analysis of variance and mean comparisons using the Tukey test, with *p* < 0.05 adopted as the significance criterion for F and for differences between means. For the time factor, regressions were fitted, and the models were selected based on the significance of the regression coefficients using the ‘t’ test at a 5% probability level. The data were processed using the software SISVAR 5.1 (Ferreira, 2000) and SIGMAPLOT 10.0 (Systat Software Inc.).

## Results and discussion

The reference area showed a total average of 983.92 mg kg^−1^ of REE and 66.84 mg kg^−1^ of U and Th in the soil profile up to a depth of 2 m. The REE distribution comprised 87.63% of LREE and 12.37% of HREE. Regarding actinides, the concentrations showed a predominance of Th (73.26%) compared to U (26.74%). These results corroborate the mineralogical composition expected for the kimberlites intrusions in the area (Benitez, 2009; Chaves et al., 2008) as well as the presence of minerals such as titanite and apatite in the soil of the region (Fig. S2). Furthermore, a homogeneity in the elements’ distribution can be observed across the seven depths, confirming the geogenic origin of these elements in the soil (Table 4).

**Table 4:** Concentration and average increase of REE, U, and Th in 28 t ha^−1^ of Araxá phosphogypsum applied

Sc	Y	La	Ce	Pr	Nd	Sm	Eu	Gd	Tb	Dy	Ho	Er	Tm	Yb	Lu	Th	U	Total
Araxá phosphogypsum																		
mg kg^−1^																		
1.59	77.11	819.10	1505.57	194.19	712.79	102.88	26.51	94.91	8.23	29.16	3.85	9.02	0.67	2.90	0.31	13.89	1.31	3604.00
Estimated addition*																		
mg m^−1^ (linear)																		
11.12	539.78	5733.72	10,539.01	1359.36	4989.53	720.16	185.55	664.36	57.60	204.10	26.97	63.11	4.72	20.33	2.14	97.25	9.18	25,227.98
mg kg^−1^ of soil																		
0.74	35.99	382.25	702.60	90.62	332.64	48.01	12.37	44.29	3.84	13.61	1.80	4.21	0.31	1.36	0.14	6.48	0.61	1681.87

^*^Estimated values for layer 0–5 cm, calculated based on concentrations found in phosphogypsum samples from Araxá

The phosphogypsum derived from Araxá apatite contains a total concentration of REE, Th and U of 3.60 g kg^−1^ (Table 4). This value corresponds to 95.89% of LREE, 3.69% of HREE, and 0.42% of U, and Th. Similar values were found in studies conducted with phosphogypsum from the city of Tapira (Dinali et al., 2019; Moreira, 2014; Oliveira, 2012; Ramos et al., 2016a, 2016b). Qing et al. (2025) also reported an overall REE content in phosphogypsum of about 0.49 wt%, dominated by La, Ce, Pr, and Nd, while Dy, Eu, Nd, Tb, and Y together accounted for a considerable proportion of 26.2 wt%. Several authors (Aide & Pavich, 2002; Tyler, 2004a) have already reported the presence of REE, U, and Th in phosphate minerals, with their concentrations depending on the origin and genesis of the phosphates (Picard et al., 2002).

The estimated addition of Ce by phosphogypsum was 702.60 mg kg^−1^ (Table 4), which means approximately seven times the background values in the reference area (Table 3), while for Th, the addition was similar to the natural contents (6.48 mg kg^−1^ and 7.00 mg kg^−1^, respectively), and U received a contribution smaller than the reference concentration (0.61 mg kg^−1^ and 2.55 mg kg^−1^, respectively). This indicates that each element is added in different proportions relative to background levels, potentially altering chemical equilibria, particularly for each individual element.

The concentration of Th corresponds to 91.38% of the total actinide content in the phosphogypsum. Different origins of phosphates can lead to variations in geochemical abundances, as Th levels in sedimentary phosphates are much lower than in those of igneous origin (Akfas et al., 2024). It is worth noting that the phosphogypsum from Araxá, used in the present study, comes from apatite of igneous origin.

The samples from the reference area, which had no phosphogypsum application, showed concentrations of 409.96 mg kg^−1^ for the sum of REE and 28.4 mg kg^−1^ for the sum of U and Th at the 0–20 cm depth (Table 4). According to Tyler (2004a), Liang et al. (2005), and Hu et al. (2006), the concentrations of REE in preserved soils show minimum and maximum values of 16 and 700 mg kg^−1^, respectively, with an observed average of 165 mg kg^−1^. For Brazilian soils, Paye et al. (2016) reported a sum of REE of 100.81 mg kg^−1^, while Amazonian Soils specifically showed values between 82.93 mg kg^−1^ and 35.36 mg kg^−1^ (Ferreira et al., 2021). Therefore, the values obtained in this study can be considered relatively high, which is attributed to the parent materials of the soils in the Alto São Francisco region.

**Fig. 1:**
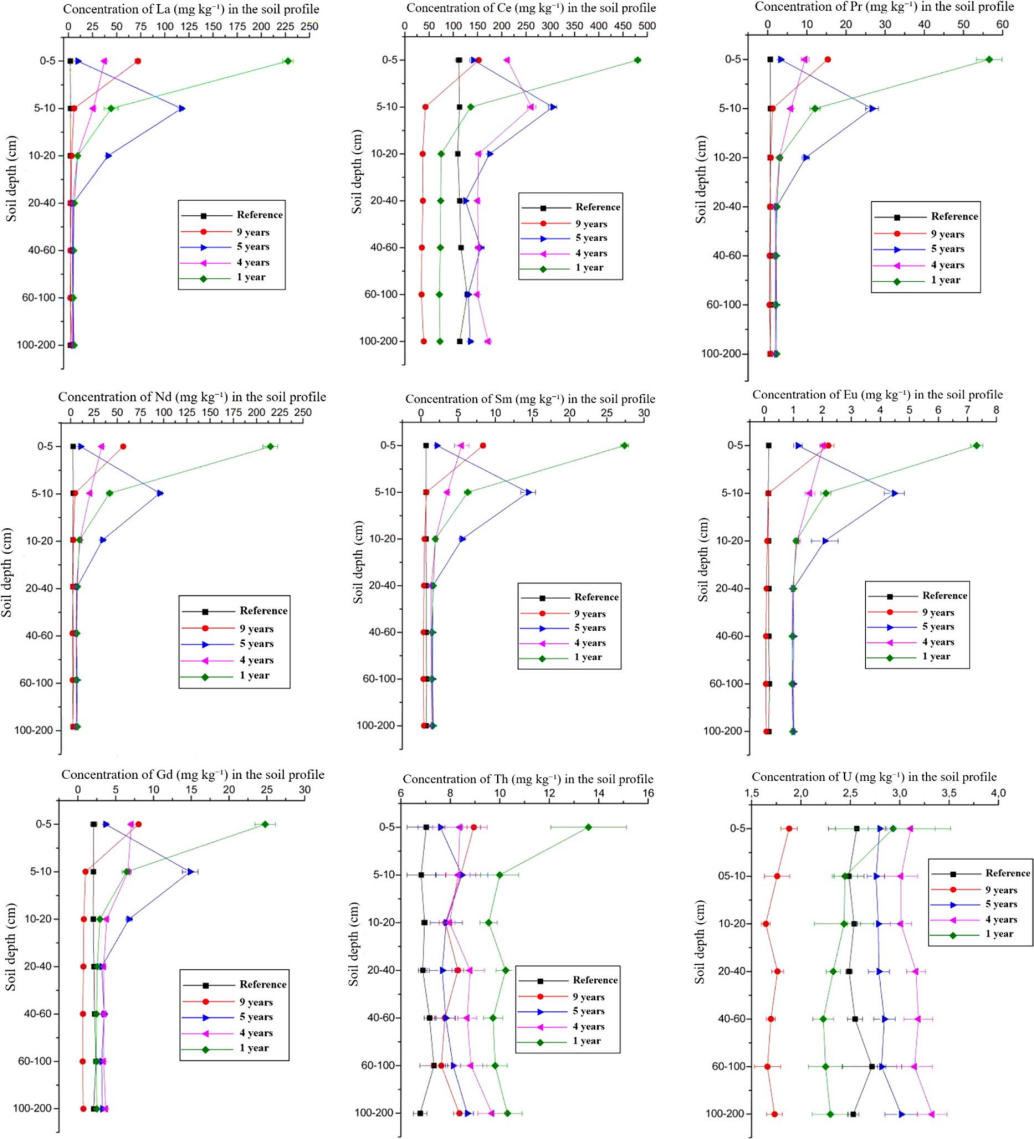
Distribution of LREE, U, and Th in the soil profile in areas with phosphogypsum application and reference area over the evaluated years

**Fig. 2:**
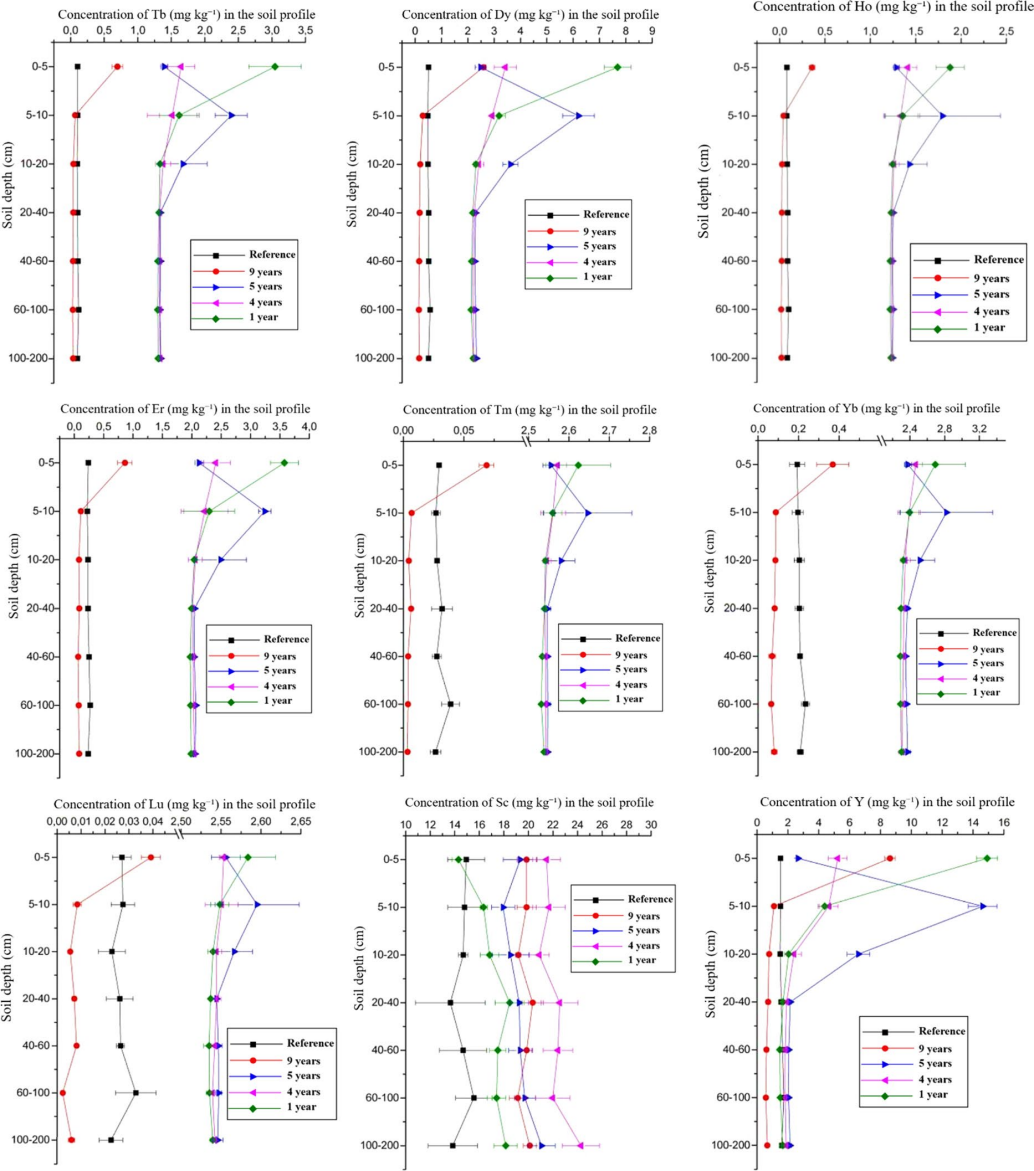
Distribution of HREE in the soil profile in areas with phosphogypsum application and of the reference area over the evaluated years

The application of 7 kg m^−1^ of Araxá phosphogypsum to the soil surface (0–5 cm layer) resulted in a substantial increase in the inputs of REE, U, and Th, reaching a total addition of 1681 mg of elements per kg of soil (Table 4). The overall behavior of REE, U, and Th can be seen in Figs. 1 and 2, and as expected, the surface layer (0–5 cm) exhibited the highest concentrations of REE, U, and Th, primarily due to the persistence of undissolved phosphogypsum, especially at 1 and 9 years after the application. Additionally, the concentrations observed 9 years after application are closer to the reference values. These findings indicate that the time elapsed since application was insufficient for complete dissolution, given the large volume applied. Thus, a period longer than 9 years would be required for all phosphogypsum residues to be totally leached.

In addition to incomplete dissolution, other factors contributed to the accumulation of these elements in the surface layers, such as the high clay and organic matter contents, which are associated with increased effective CEC (Tables 1 and 2), as noted by Laveuf and Cornu (2009). Importantly, there was likely leaching of the natural REE contents, as the soils that received phosphogypsum showed lower concentrations of these elements in the subsurface compared to the reference soil nine years later. This may be attributed to the chemical inputs associated with the phosphogypsum application, which likely enhanced the mobilization of chemical elements. All elements showed regression model adjustments for the evaluated times, except for Y, which presented a high coefficient of determination (Figs. S3 and S4). Numerical values and statistical comparisons are shown in Tables S3 and S4.

According to Lima & Ottosen (2021), the primary sinks for REE in soils include organic complexes, manganese oxides, and iron (Fe) oxyhydroxides. Silicate clay minerals also play an important role in REE retention, acting as carriers, especially in more weathered soil profiles (Gnandi & Tobschall, 2003; Öhlander et al., 1996; Papoulis et al., 2004). Thus, clay minerals are key contributors to the control of REE stocks in soils (Xing & Dudas, 1993). Also, the extent to which REE bind to organic matter depends on the type, composition, and quantity of organic matter present, as well as on soil pH and redox conditions (Grybos et al., 2007). Due to its high density of negative charges per unit dry weight, organic matter can show a strong ability to complex and adsorb REE (Pourret et al., 2007; Tyler, 2004a, 2004b).

The soil that received phosphogypsum nine years prior to sampling contained the highest levels of organic matter and showed a smaller variation among soil depths, as indicated by the observation that even at 200 cm depth, 3% organic matter was present. This may explain the elevated REE contents in the surface layer relative to the reference soil. Even 9 years after phosphogypsum application, the organic matter content can contribute to the retention of REE in the surface.

In most cases, the concentration of REE, U, and Th in the reference area was lower compared to the area that received phosphogypsum, and as the depth increases, this difference decreases. For instance, regarding Th levels, reference soil showed lower values down to 200 cm (6.78 mg kg^−1^) compared to treated soils, with the highest concentrations observed in the area sampled one year after application (10.38 mg kg^−1^). In contrast, La levels in all treatments and reference area were close to each other since 20 cm, showing no statistical difference between years and from this soil depth (*p* < 0.05).

The most abundant chemical element evaluated in the treated areas was Ce. Its levels in the reference area, especially down to 20 cm in the years 2005 and 2013 (9 and 1 year after application), were greater than in treated soil, suggesting that the background levels in that area are higher than expected. In general, Ce stands out as the predominant REE in soils according to various studies on soil background characterization (Bispo et al., 2021; Ferreira et al., 2021). In fact, Ce was the most abundant chemical element in the reference area. It is the only lanthanide that can occur in both the oxidized states Ce^3+^ and Ce^4+^, which gives this element lower mobility in its tetravalent state compared to the trivalent one (Smedley, 1991). The lower solubility of Ce relative to the other LREE can lead to its enrichment in some cases, a phenomenon known as a positive Ce anomaly, commonly observed in many REE reserves (Luo et al., 2024).

### Light rare earth elements

In general, there was a reduction of the concentration of LREE over time in the 0–40 cm layer. The greatest movement of these chemical elements in these soil layers is due to the higher concentration of SO_4_^2−^, which is very soluble, and once applied to the soil, phosphogypsum partially dissociates, releasing SO_4_^2−^, which forms ionic pairs. The REE, as simple sulfates, this is, REESO_4_^+^ (Migdisov & Williams-Jones, 2008; Migdisov et al., 2006), remain in the soil solution (Tyler, 2004a) and can be leached through their association with simple or double sulfates of variable composition. This occurs because lanthanides are classified as hard acids (Aide & Aide, 2012), which preferentially coordinate with hard bases, particularly those containing oxygen, nitrogen, and sulfur as donor atoms.

Specifically in the 0–5 cm layer, it was observed that, compared to the reference area, chemical elements were differently concentrated one year after application. La increased to levels up to 100 times higher than the reference area one year after application, Ce four times, Praseodymium (Pr) 80 times, Neodymium (Nd) 100 times, Samarium (Sm) 40 times, Europium (Eu) 50 times, Gadolinium (Gd) 12 times, and Th to twice the reference values. In contrast, U concentrations showed almost no change between 2013 and 2014.

Among the subsurface layers (20–200 cm), no significant differences in element concentrations were observed across depths or time periods. These results suggest that within six years following phosphogypsum application, LREE were redistributed throughout the soil profile down to 2 m, or leached beyond the sampled depth. The fact that LREE concentrations in the surface approached those of the reference area after 9 years of application indicates that no deposition occurred within the profile at greater dephts. Therefore, the evidence points toward downward leaching to deeper layers, raising concerns about the potential risk of groundwater contamination.

This lack of enrichment downward the soil profile for LREE compared to the surface means that the sorption process was not sufficient to retain these elements at the subsurface. Adsorption and desorption of REE on clay minerals are dependent on pH (Aja, 1998; Bishop et al., 2024; Gutierrez-León et al., 2025; Kachoueiyan et al., 2024; Wen et al., 2002). Cao et al. (2001) observed gradual leaching of REE as the soil pH decreased from 7.5 to 3.5, with the effects being more pronounced for the elements La, Ce, Gd, and Y. According to Bishop et al. (2024), clay type and ionic strength are also important in the REE sorption processes. Considering that the highest pH of soil samples were 5.95 and the lowest 4.65 (Table 1) and the mineralogy was based on kaolinite and Fe and Al oxides, typical from weathered soils, it is reasonable to assume that the amount of negative charges available to retain these elements was insufficient, which is in agreement with the low CEC in the subsurface layers of the soil (Table 1).

The mobility of REE is related to their respective ionic radii (Bregiroux et al., 2006; Laveuf & Cornu, 2009), a trend that was observed for LREE in this study. When evaluating the depletion rates of the elements La (1.03 Å); Pr (0.99 Å); Sm (0.96 Å); and Eu (0.94 Å), comparing their contents in the years 2005 (9 years after application) and 2013 (1 year after application) for the 20–200 cm layer, there was a decrease of 51%, 74%, 76% and 93%, respectively, between years. The values observed in the 0–20 cm layer were not considered to avoid the influence of potential residual phosphogypsum on this comparison. Thus, depletion becomes more pronounced as atomic radii decrease, indicating that ions with smaller hydrated radii are preferentially adsorbed in soils relative to larger ones after a chemical input. This fact also corroborates the similar behavior of LREE ascribed to the lanthanide contraction (Aide & Aide, 2012). This behavior results from the progressive filling of the 4f orbitals in the antepenultimate electron shell, while all LREE maintain a 6s^2^ configuration in their outermost shell, classifying them as inner transition metals (Kanazawa & Kamitani, 2006).

### Uranium and Thorium

The results showed that U exhibited low mobility in the soil over the years, without showing significant differences between the studied layers (Fig. 1). The only area showing a statistical difference (*p* < 0.05) was the one that received phosphogypsum one year prior to sampling (2013), where the surface layer presented higher concentrations (Table S3). The area with the phosphogypsum application in 2005 showed lower concentrations compared to the reference area, suggesting that the background content of U is already high, likewise for Ce, a consequence of the local geology. Furthermore, it may indicate that 9 years was sufficient time to a total leaching of the applied U, since no enrichment was observed in deeper soil layers. It should be noted that the contribution of phosphogypsum to U in the soil is small compared to the other elements, amounting to only 0.61 mg kg^−1^. From 2013 to 2005, a 24% decrease in U content was observed in the subsurface (20–200 cm).

The application of phosphogypsum increased Th concentrations in the soils compared to the reference area. The areas that received phosphogypsum applications showed higher total concentrations along the soil profile, especially at the surface, with increases that reached 93% in 2013. Th concentrations decreased over time following phosphogypsum application, with the greatest reductions in the surface layers (0–20 cm) and a slight tendency for accumulation, though non-significant at greater depths.

The accumulation percentages demonstrate rapid depletion of Th over time throughout the entire profile, especially between the application years of 2013 and 2010. Attention is drawn to the Th concentrations in the 100–200 cm layer of the treated area in 2013. This area showed an increase of 3.58 mg kg^−1^ after phosphogypsum application, representing a 51.91% increase in Th concentration compared to the reference area, gradually lost over the years. Although Th is generally considered a relatively immobile element, Santofimia et al. (2022) reported that the formation of soluble complexes with sulfates, nitrates, carbonates, phosphates, silicates, and organic compounds can increase its solubility and mobility under natural conditions.

### Heavy rare earth elements

Similar to the LREE, the HREE also exhibited higher concentrations in the areas that received phosphogypsum compared to the reference area. However, the significant variations in the concentrations of HREE were more limited to the surface layers (0–10 cm) and were lower compared to the LREE for all areas with phosphogypsum application. Additionally, HREE concentrations in the 20–200 cm layers showed significant differences only for the area with the longest time since phosphogypsum application (2005) compared to the others.

The chemical elements Y, Tb, Ho, and Ytterbium (Yb) revealed a decrease from 2013 to 2005 of 59%, 98%, 98% and 97%, respectively, following the decrease of atomic radii. This is in agreement with the tendency also observed in LREE, in which larger ionic radii correspond to smaller hydrated radii and are more adsorbed and less mobile after such chemical input in soil.

Lu, Thulium (Tm), and Yb revealed greater concentrations at the reference area compared to the area that received phosphogypsum in 2005 at the surface layer, indicating a total loss of these added elements. This is consistent with the lack of enrichment of these elements relative to the reference area, which may also reflect the high background of these soils.

When the normalized concentrations of LREE and HREE (according to the reference samples) were evaluated across all soil depths, it was observed that the ΣLREE/ΣHREE ratio increased from 0.33 (1 year after application) to 2.67 (9 years after application), indicating a relative enrichment of LREE compared to HREE. These findings are attributed to differences in the behavior of LREE and HREE in the soil solution (Braun et al., 1998; Panahi et al., 2000; Tyler, 2004a), which result in greater or lesser adsorption of these elements by the exchange complex. This pattern is consistent with observations in natural soils, where HREE can be preferentially leached (Mihajlovic & Rinklebe, 2018). This result is also due to the input of LREE and HREE in the phosphogypsum, since from 1681.87 mg kg^−1^ of total REE added (Table 4), 95.89% is LREE, which means that the greatest input of REE from the phosphogypsum is of LREE.

### Dynamics of increments and losses of REE U and Th with the application of phosphogypsum

The movement dynamics of REE, U, and Th in the soil profiles can be addressed through the increments and losses of these elements over time. For this purpose, the natural concentrations (Cr) of these elements in the reference soil, the theoretical inputs (I) calculated from the contribution of applied phosphogypsum, and the observed concentrations in the soils (Co) after phosphogypsum application were considered (Table 5). Another figure was included showing only the Co/Cr ratio, to assess the influence of phosphogypsum on enrichment and depletion patterns (Fig. S5). Values greater than one indicate enrichment (green), whereas values lower than one indicate depletion (red).

**Table 5:** Dynamics of increases and losses of LREE’s, U, and Th over time in the soil profile

Depth (cm)	LREE, U and Th											
	2005	2008	2010	2013	2005	2008	2010	2013	2005	2008	2010	2013
	mg kg^−1^											
	La				Ce				Pr			
0–5	− 312.28^*^	− 374.65	− 346.66	− 156.55	− 661.44	− 672.73	− 602.39	− 333.61	− 75.95	− 88.00	− 81.70	− 34.70
5–10	3.65	114.99	23.63	42.10	− 70.29	192.23	148.77	29.40	0.53	25.92	5.22	11.39
10–20	1.22	39.00	7.18	7.36	− 72.29	65.82	42.53	− 34.06	0.09	9.04	2.38	2.39
20–40	0.54	2.82	2.23	4.08	− 75.99	11.71	36.94	− 39.40	− 0.06	1.55	1.33	1.69
40–60	− 0.06	2.40	1.95	2.98	− 80.75	40.59	35.98	− 42.41	− 0.21	1.47	1.21	1.39
60–100	− 0.40	2.39	1.88	2.53	− 94.20	1.45	20.90	− 57.22	− 0.28	1.48	1.21	1.36
100–200	1.74	3.05	2.42	3.79	− 74.21	21.78	58.75	− 40.58	0.06	1.62	1.33	1.64
Σ	− 305.58	− 209.99	− 307.36	− 93.70	− 1129.17	− 339.15	− 258.52	− 517.88	− 75.82	− 46.92	− 69.02	− 14.84

	Nd				Sm				Eu			
0–5	− 278.69	− 324.60	− 301.62	− 120.32	− 40.35	− 46.55	− 43.22	− 21.32	− 10.32	− 11.37	− 10.49	− 5.21
5–10	2.04	93.26	18.18	39.22	0.04	13.73	2.86	5.60	− 0.01	4.33	1.43	1.98
10–20	0.33	31.94	7.60	7.08	− 0.20	4.85	1.27	1.24	− 0.04	1.94	0.97	0.95
20–40	0.29	4.75	4.16	5.03	− 0.31	0.91	0.69	0.96	− 0.09	0.84	0.82	0.84
40–60	− 0.78	3.69	3.23	3.83	− 0.36	0.87	0.68	0.78	− 0.10	0.84	0.80	0.80
60–100	− 0.78	4.21	3.31	3.55	− 0.44	0.81	0.64	0.70	− 0.12	0.82	0.80	0.78
100–200	0.08	4.54	3.54	4.59	− 0.30	0.96	0.75	0.88	− 0.08	0.85	0.83	0.83
Σ	− 277.51	− 182.21	− 261.60	− 57.02	− 41.91	− 24.41	− 36.32	− 11.15	− 10.76	− 1.75	− 4.84	0.97

	Gd				U				Th			
0–5	− 38.37	− 42.70	− 39.31	− 21.63	− 1.31	− 0.39	− 0.07	− 0.25	− 4.57	− 5.92	− 5.13	0.07
5–10	− 1.04	12.78	4.56	4.39	− 0.90	− 0.85	− 1.00	0.68	1.59	1.64	1.49	3.17
10–20	− 1.25	4.74	1.79	0.89	− 1.69	− 1.66	− 1.53	0.06	0.84	0.87	1.00	2.59
20–40	− 1.40	1.03	1.29	0.45	− 1.71	− 1.08	− 0.59	0.85	0.78	1.41	1.90	3.34
40–60	− 1.57	1.12	1.23	0.25	− 1.93	− 1.90	− 1.03	0.01	0.62	0.65	1.52	2.56
60–100	− 1.75	0.75	1.01	0.07	− 2.43	− 1.95	− 1.23	− 0.25	0.29	0.77	1.49	2.47
100–200	− 1.38	1.14	1.58	0.41	− 0.95	− 0.63	0.37	0.99	1.58	1.90	2.90	3.52
Σ	− 46.76	− 21.14	− 27.85	− 15.17	− 10.91	− 8.45	− 5.07	2.10	1.13	1.32	5.17	17.72

	HREE											
	Sc				Y				Tb			
0–5	4.16	3.65	5.80	− 1.38	− 28.89	− 34.84	− 32.27	− 22.59	− 3.25	− 2.55	− 2.30	− 0.90
5–10	5.05	3.14	6.89	1.56	− 0.43	13.10	3.12	2.86	− 0.04	2.29	1.41	1.51
10–20	4.47	3.85	6.19	2.15	− 0.72	5.07	0.89	0.54	− 0.07	1.57	1.27	1.23
20–40	6.70	5.60	8.90	4.83	− 0.87	0.57	0.33	0.10	− 0.07	1.23	1.22	1.21
40–60	5.17	4.65	7.72	2.82	− 1.05	0.39	0.16	− 0.19	− 0.08	1.22	1.21	1.19
60–100	3.57	4.16	6.42	1.85	− 1.17	0.31	0.08	− 0.23	− 0.09	1.21	1.20	1.17
100–200	6.26	7.22	10.44	4.30	− 0.94	0.54	0.28	0.07	− 0.07	1.24	1.23	1.20
Σ	35.38	32.27	52.36	16.13	− 34.07	− 14.86	− 27.41	− 19.44	− 3.67	6.21	5.24	6.61

	Dy				Ho				Tm			
0–5	− 11.52	− 11.62	− 10.69	− 6.43	− 1.53	− 0.60	− 0.47	− 0.01	− 0.28	2.21	2.23	2.28
5–10	− 0.20	5.72	2.44	2.70	− 0.04	1.71	1.25	1.27	− 0.02	2.61	2.53	2.52
10–20	− 0.31	3.13	1.92	1.81	− 0.06	1.35	1.17	1.16	− 0.02	2.55	2.51	2.51
20–40	− 0.35	1.78	1.73	1.68	− 0.07	1.16	1.15	1.14	− 0.03	2.51	2.50	2.50
40–60	− 0.36	1.75	1.68	1.63	− 0.07	1.15	1.14	1.13	− 0.02	2.51	2.51	2.50
60–100	− 0.43	1.72	1.62	1.55	− 0.09	1.15	1.13	1.11	− 0.04	2.50	2.50	2.49
100–200	− 0.36	1.81	1.72	1.69	− 0.06	1.16	1.15	1.14	− 0.02	2.51	2.51	2.50
Σ	− 13.53	4.29	0.42	4.63	− 1.92	7.08	6.52	6.94	− 0.44	17.41	17.30	17.31

	Er				Yb				Lu			
0–5	− 3.60	− 2.33	− 2.05	8.02	− 1.19	0.82	0.91	1.14	− 0.13	2.38	2.38	2.41
5–10	− 0.12	3.02	1.99	2.07	− 0.11	2.61	2.18	2.19	− 0.02	2.56	2.52	2.51
10–20	− 0.16	2.26	1.82	1.81	− 0.12	2.31	2.14	2.12	− 0.02	2.54	2.52	2.52
20–40	− 0.15	1.81	1.78	1.76	− 0.12	2.17	2.12	2.09	− 0.02	2.51	2.51	2.50
40–60	− 0.19	1.78	1.77	1.72	− 0.15	2.14	2.11	2.08	− 0.02	2.51	2.51	2.50
60–100	− 0.20	1.79	1.75	1.71	− 0.17	2.13	2.08	2.06	− 0.03	2.51	2.51	2.50
100–200	− 0.15	1.82	1.81	1.75	− 0.14	2.16	2.10	2.09	− 0.02	2.52	2.52	2.51
Σ	− 4.58	10.14	8.86	18.82	− 2.00	14.34	13.64	13.77	− 0.25	17.53	17.47	17.45

^*^Values for the 0–5 cm layer refer to the difference (d):d = [Co–(Cr + I)], where Co is the concentration obtained; Cr is the concentration in the reference area and I is the input from the use of phosphogypsum for the element considered. For the other layers, the results are the difference: d = (Co–Cr). Positive values mean accumulations and negative values mean losses

The results reveal an increase in losses or a decrease in increments over time, which is expected with the leaching process.

When considering the input of phosphogypsum, almost all chemical elements reveal a loss pattern in the surface layer, except for Sc, Tm, Yb, and Lu, all HREE (Table 5). According to the enrichment factor calculated by the concentration found normalized to the reference area (Fig. S5 and S6), these elements exhibited enrichment in the surface layer. However, although the LREE tended to concentrate along the soil profile, they showed substantial leaching when the amount of phosphogypsum applied was taken into account. For example, by 2005, La showed losses of 305.58 mg kg⁻^1^, while Ce showed losses of 1129.17 mg kg⁻^1^. This is attributed to the great abundance of these elements in the phosphogypsum and in the reference soil.

In soils naturally enriched with REE, a fractionation towards the leaching of HREE is observed, since they possess a smaller ionic radius and stronger hydrolysis ability when compared to LREE (Roy et al., 2024). This finding is consistent with previous studies in soil profiles (Mihajlovic & Rinklebe), but inconsistent with others (Ramos et al., 2016b) and the present study demonstrates that the application of exogenous materials to the soil can modify these dynamics. However, a depletion pattern was clearly observed in the HREE after 9 years, compared with the reference area.

Sc revealed one of the greatest gains due to the phosphogypsum application in the surface (Table 5), consistent with the enrichment observed relative to the reference area (Fig. S5). Along with Th, both elements are highly abundant in the reference soil, only less abundant than Ce, for which phosphogypsum application also promoted gains of up to 17.72 mg kg⁻^1^ one year after. However, this gain was reduced to 1.13 mg kg⁻^1^ in 2005. The behavior of Th appears to be relatively immobile, as previous studies have indicated that the available fraction of Th in tropical soils after application of phosphogypsum is low (Nisti et al., 2015).

Among the HREE, Y exhibited greater loss due to the phosphogypsum application, because its concentration in the byproduct is the highest among the HREE. U showed losses following phosphogypsum application (Table 5), and compared to the reference area (Fig. S5), depletions were evident both in 2013 and 2005. U contamination in soil is particularly concerning, as up to 50% of it can be leached from the topsoil within the first year of contamination in a field experiment (Rout et al., 2022).

## Conclusion

Despite its high solubility, phosphogypsum, or more precisely, the impurities resulting from its dissolution, such as REE, can persist in the soil for extended periods. This underscores the importance of applying byproducts in a rational and controlled manner. Even materials widely considered suitable for agricultural use can significantly influence the dynamics of chemical elements in the soil.

This study demonstrates the mobility of REE triggered by phosphogypsum application in a widespread tropical soil. Although REE are often treated as a chemically homogeneous group, the results revealed notable differences in their behavior. This finding reinforces the need to analyze REE at least as two distinct subgroups: LREE and HREE. Among the REE, LREE exhibited the highest concentrations, with Ce being the most abundant. A general decline in REE concentrations was observed over time, however, the influence of REE inputs from phosphogypsum application is still evident nine years later.

The application of phosphogypsum can increase LREE concentration in surface soils, with progressive enrichment observed towards elements with larger atomic radius, whereas HREE occurred at lower concentrations and showed a smaller magnitude of enrichment. Over the years, the normalized ratio ΣLREE/ΣHREE increases, indicating a relative enrichment of LREE. U and Th also exhibited contrasting behaviors due to phosphogypsum application at the surface. Th tended to accumulate throughout the profile, even at greater depths, resembling the behavior of less mobile REE. In contrast, U showed significant losses along the profile, raising concerns regarding deep soil contamination. This study highlights the importance of investigating REE mobilization following the application of byproducts to soil, as their behavior may differ from natural patterns observed in weathering profiles.

## Supplementary Information

Below is the link to the electronic supplementary material.

Supplementary file1 (DOCX 1642 KB)

## Data Availability

No datasets were generated or analysed during the current study.
