# Supplementary material for: Elemental redistribution in tropical soils: insights into REE, U, and Th mobility after extended phosphogypsum use
Source: Environ Geochem Health. 2026 Feb 7;48(3):146. doi: 10.1007/s10653-026-02991-6 (PMC12881094; doi:10.1007/s10653-026-02991-6)
Supplement: Supplementary file 1 — Supplementary file1 (DOCX 1642 KB) [file 10653_2026_2991_MOESM1_ESM.docx]

**Elemental Redistribution in Tropical Soils: Insights into REE, U, and Th Mobility After Extended Phosphogypsum Use**

Luís Paulo P. Tanure ^^[[1]](#footnote-1)^^, Isabela C. F. Vasques ^1*^, Renato W. Veloso ^2^, Maria Maiara C. Tanure ^3^, Walter A. P. Abrahão ^1^, Carlos Roberto Bellato^4^, Massimo Gasparon^5,6,7^, Jaime W. V. de Mello ^1^

Corresponding author: [isabela.filardi@ufv.br](mailto:isabela.filardi@ufv.br)

^1^Soils Department. Universidade Federal de Viçosa, Departamento de Solos, Avenida Peter Henry Rolfs s/n, Campus Universitário, CEP 36570-900, Viçosa - MG, Brasil.

^2^ Instituto Federal de Goiás, IFG, Águas Lindas de Goiás, 21 St., Águas Lindas de Goiás, GO, Brazil.

^3^ Instituto Federal de Educação, Ciência e Tecnologia de Mato Grosso, IFMT, Alta Floresta

Rodovia MT 208, s/n - Lote 143-A, Loteamento Aquarela - Hamoa, MT, Brazil.

^4^ Departamento de Química, Universidade Federal de Viçosa, Campus Universitário, Viçosa – MG, Brasil.

^5^ EIT Raw Materials, Knesebeckstraße 62-63, 10719, Berlin, Germany.

^6^ The University of Queensland, School of Earth Sciences, St Lucia QLD 4067, Brisbane – QUT, Australia.

^7^ INCT acqua, Universidade Federal de Minas Gerais. Av. Antônio Carlos, 6627 - Cep: 31270-901
Belo Horizonte- MG, Brasil.

**
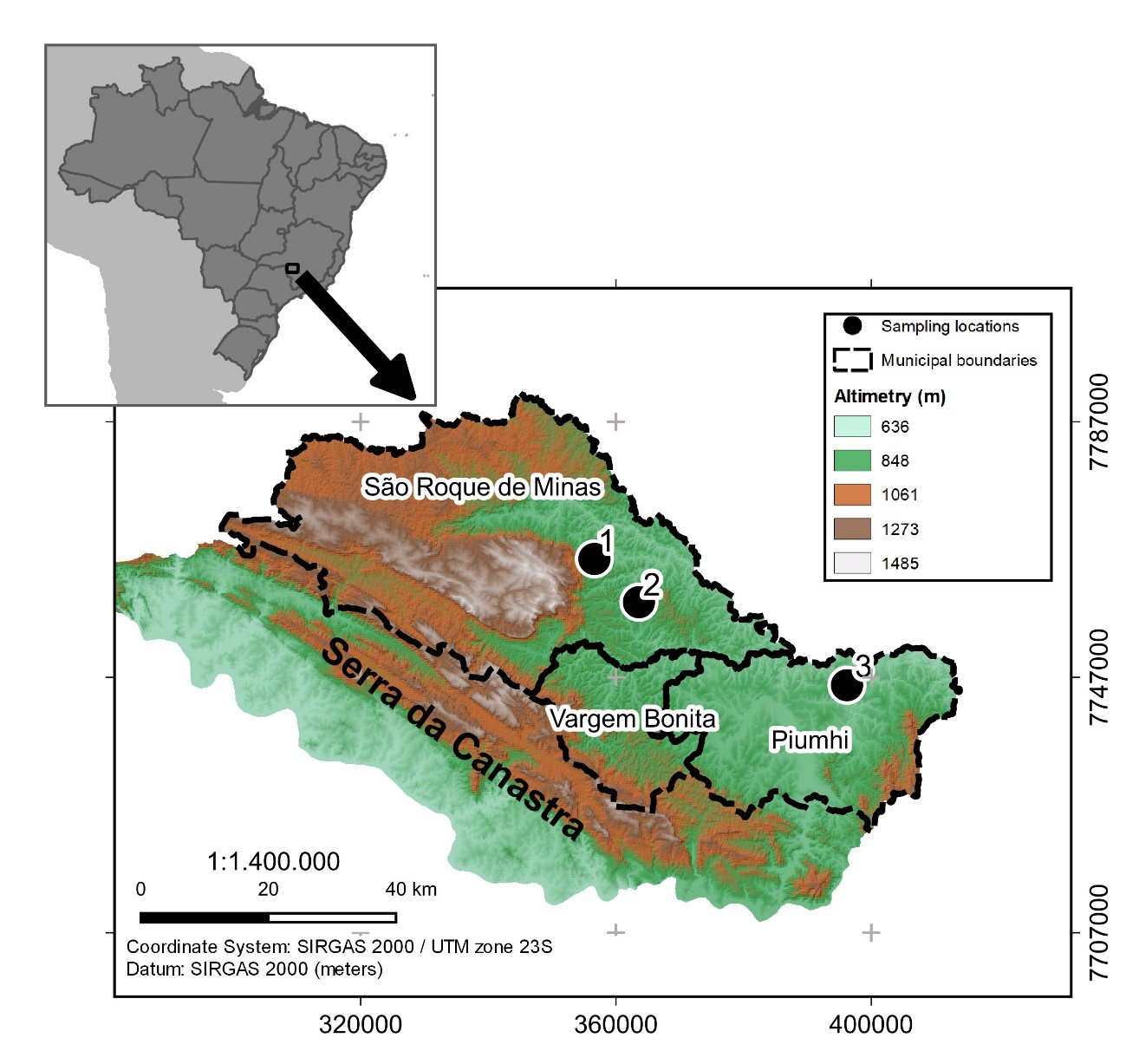
**

Figure S1: Location of the three farms where soil samples where collected.

**
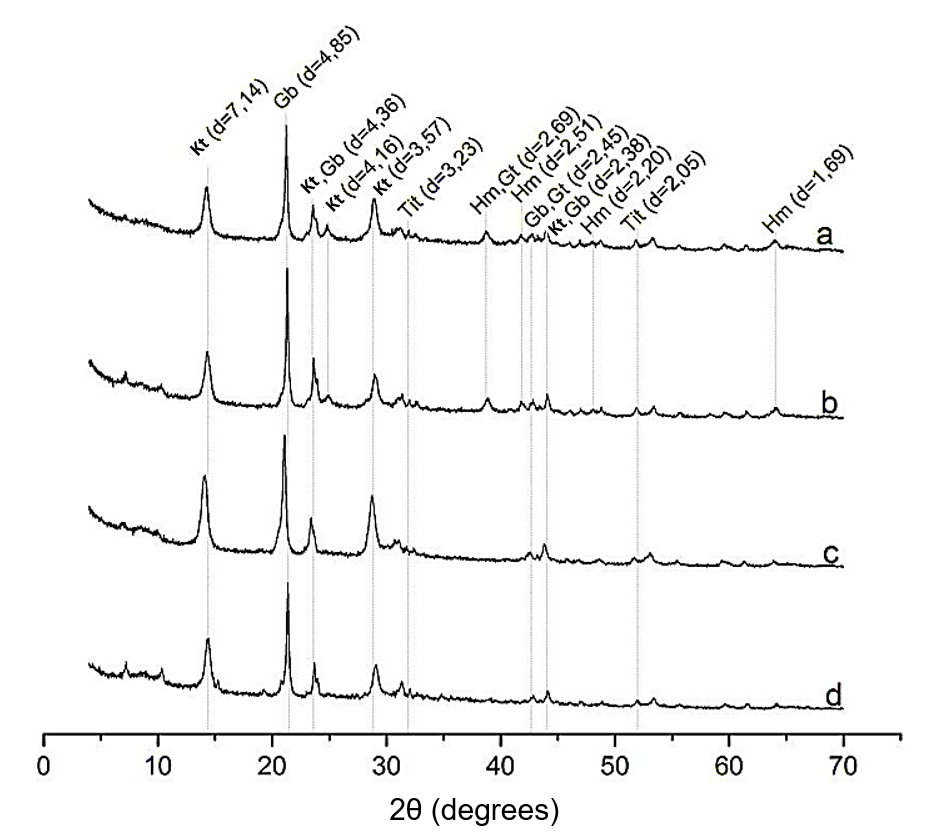
**

Figure S2: Diffractogram of depths 10-20 cm (a and c) and 100-200 cm (b and d) in natural (a and b) and deferrified (c and d) layers in dystrophic Oxisol of the study area. Ct (Kaolinite); Gb (Gibbsite); Tit (Titanite); Hm (Hematite).


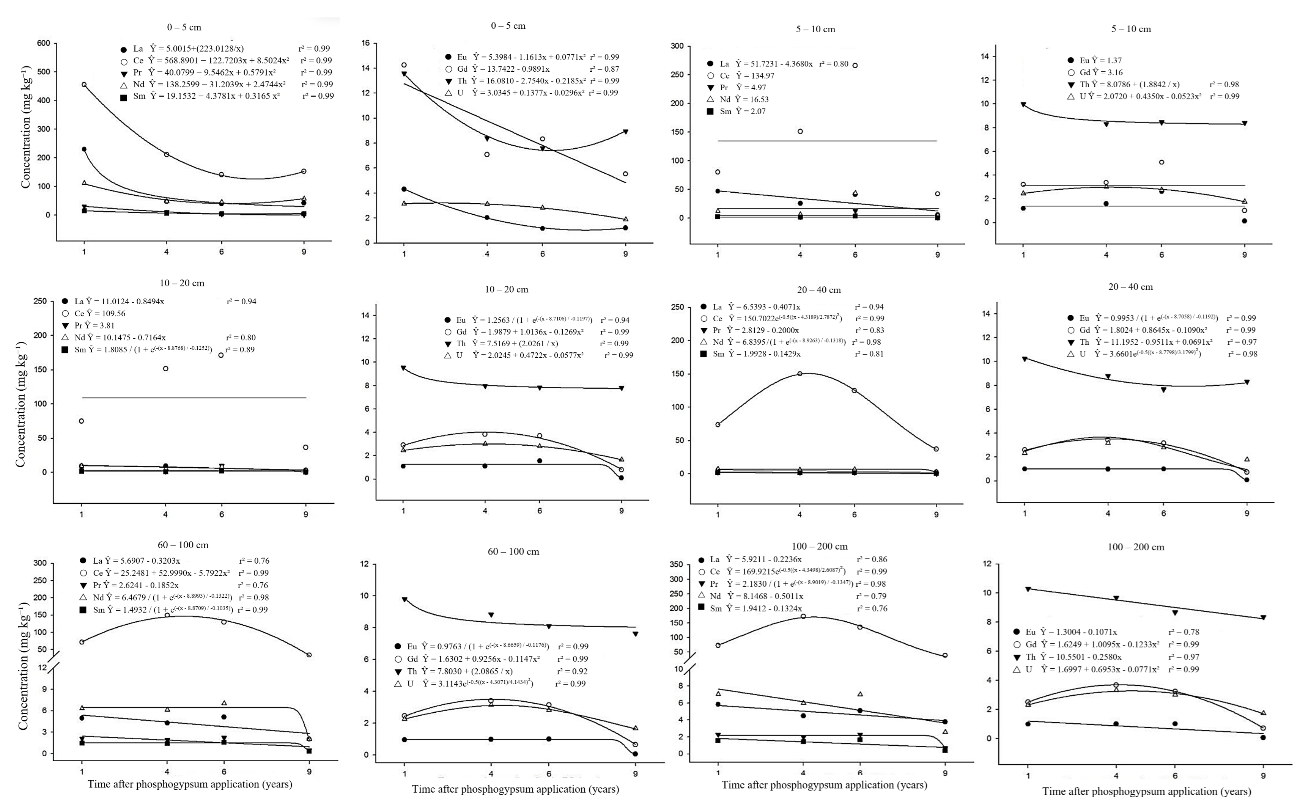


Figure S3: Concentration of REE, U and Th as a function of time after application of phosphogypsum (significant values ​​at the 5% probability level by the “t” test).


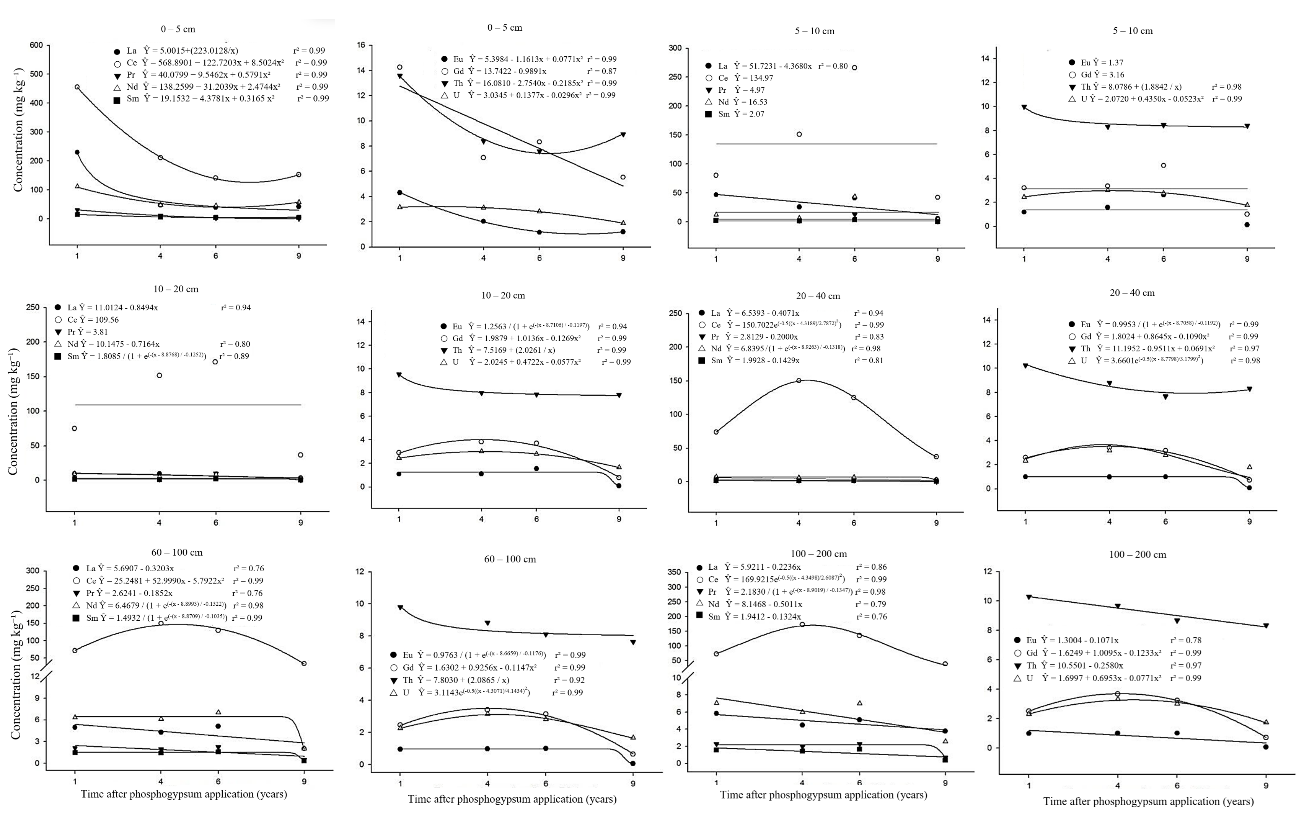


Figure S4: Concentration of REE, Sc and Y as a function of time after application of phosphogypsum (significant values ​​at the 5% probability level by the “t” test).

**
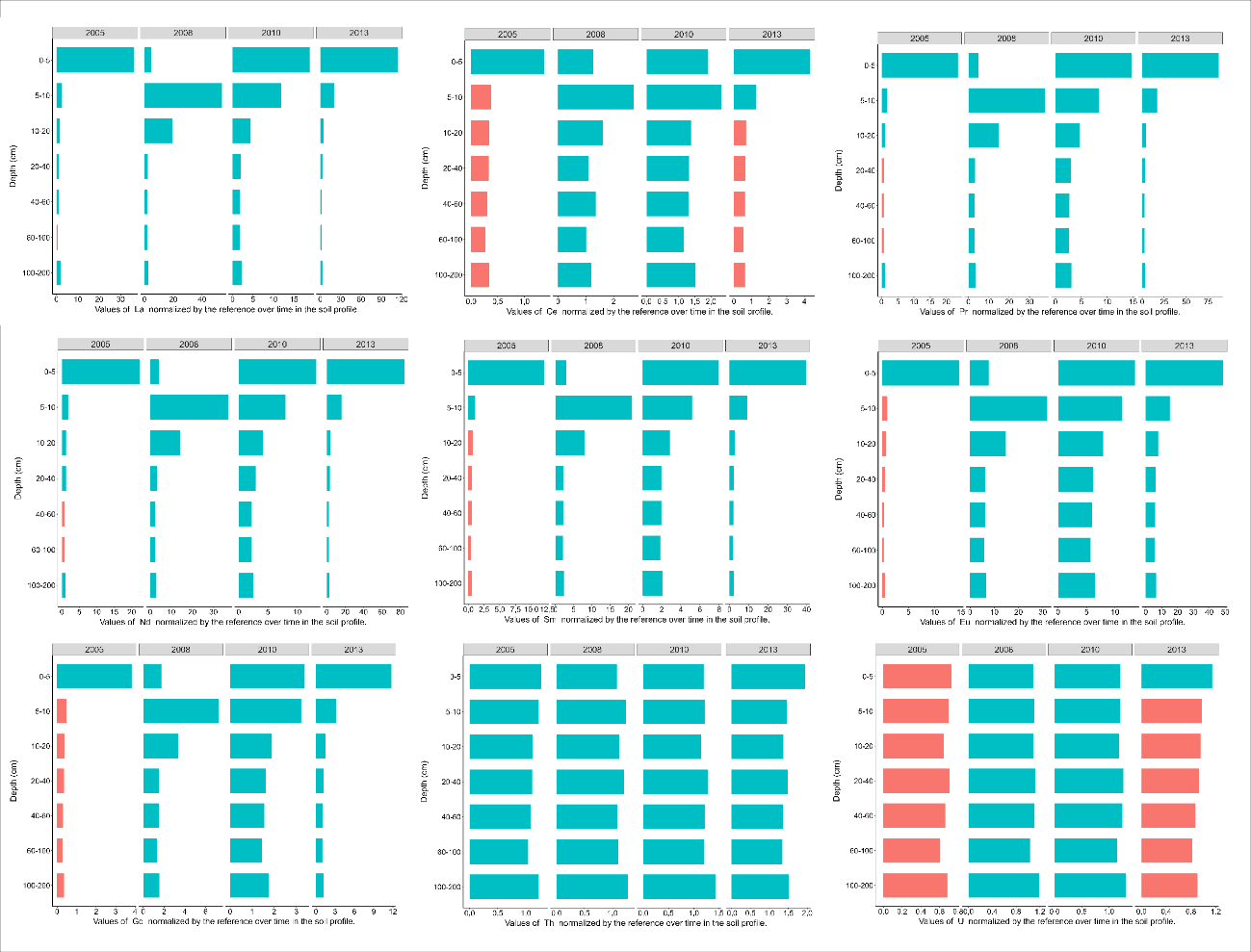
**

Figure S5: Enrichment fator ( Concentration in treated area/ Concentration in the reference area) for LREE,U and Th over the years in all soil depths. Depletions are red bards and enrichments are in green bars.

**
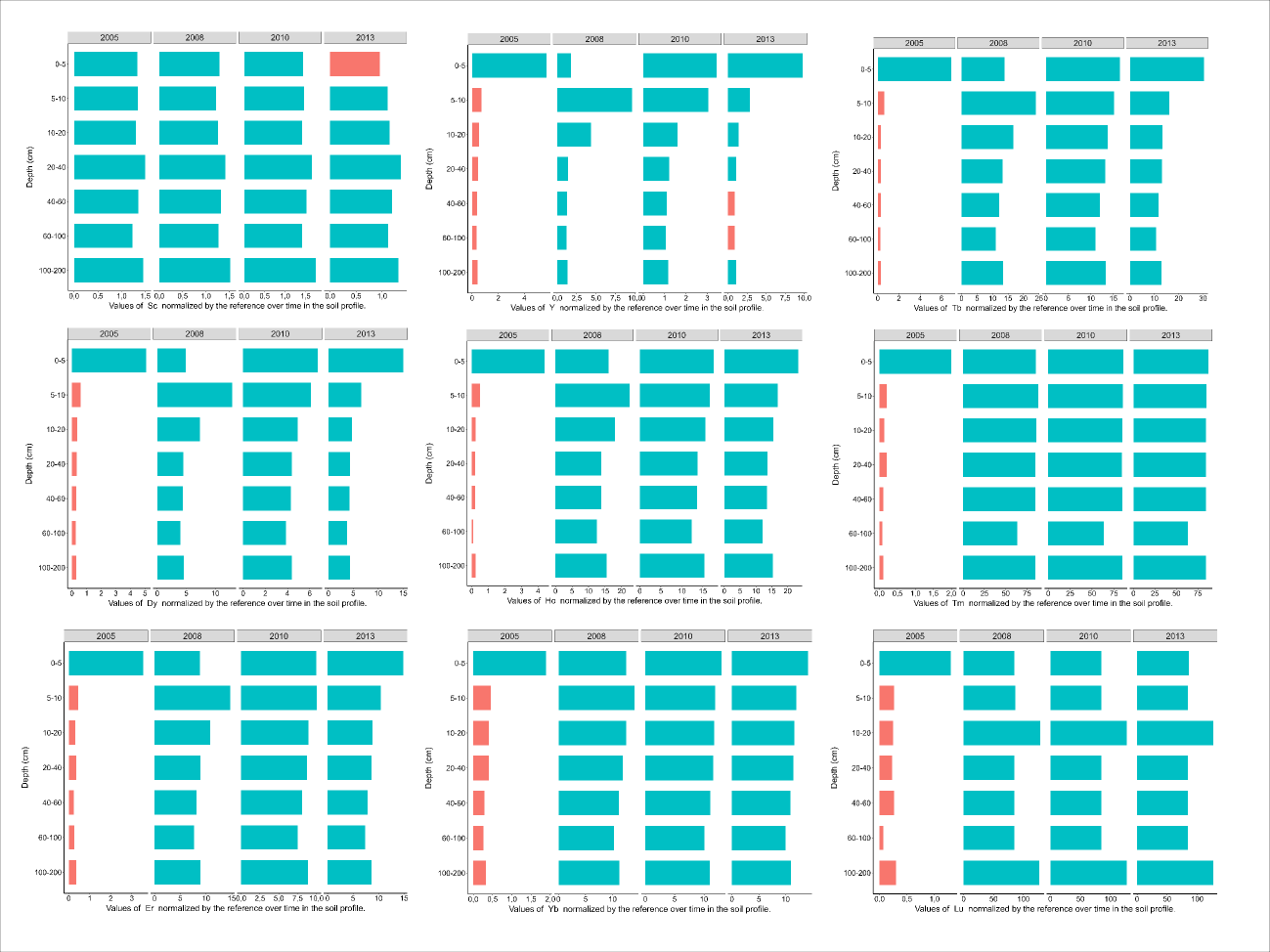
**

Figure S6: Enrichment fator ( Concentration in treated area/ Concentration in the reference area) for HREE over the years in all soil depths. Depletions are red bards and enrichments are in green bars.

Table S1. Particle density (Dp), soil bulk density (Ds), total pore volume (TPV) and hydraulic conductivity of the evaluated soil

| Horizon | Dp | Ds | TPV | Mi^(1)^ | Ma^(2)^ | Ko^(3)^ |
| --- | --- | --- | --- | --- | --- | --- |
|  | ----------------g cm^-3^--------------- | | -----------------------%---------------------- | | | cm h^-1^ |
| A | 2.52 | 1.01 | 60.0 | 42.0 | 18.0 | 10.98 |
| Bw | 2.57 | 0.93 | 64.0 | 41.0 | 23.0 | 13.13 |

^(1)^Mi: Microporosity; ^(2)^Ma: Macroporosity; ^(3)^Ko: Hydraulic conductivity (Teixeira et al., 2017).

Table S2. Detection limit (DL) and quantification limit (QL) of REE, U and Th determined in ICP-MS.

| Element | DL^(1)^ | QL^(2)^ |
| --- | --- | --- |
|  | µg L^-1^ | mg kg^-1^ |
| Sc | 3,76x10^-3^ | 1,25 |
| Y | 2,36x10^-3^ | 0,78 |
| La | 4,21x10^-3^ | 1,40 |
| Ce | 1,66x10^-2^ | 5,53 |
| Pr | 1,52x10^-3^ | 0,51 |
| Nd | 5,09x10^-3^ | 1,69 |
| Sm | 1,44x10^-3^ | 0,48 |
| Eu | 5,03x10^-4^ | 0,16 |
| Gd | 1,24x10^-3^ | 0,41 |
| Tb | 5,31x10^-4^ | 0,17 |
| Dy | 7,07x10^-4^ | 0,23 |
| Ho | 3,41x10^-4^ | 0,11 |
| Er | 7,6x10^-4^ | 0,25 |
| Tm | 4,79x10^-4^ | 0,16 |
| Yb | 6,17x10^-4^ | 0,20 |
| Lu | 3,02x10^-4^ | 0,10 |
| Th | 3,22x10^-3^ | 1,07 |
| U | 9,15x10^-4^ | 0,30 |

DL(1) = 3 σ (tgα)-1 and QL(2) = 10 σ (tgα)-1x FD. Where: σ - standard deviation of ten blank samples; α - inclination angle of the calibration curve.

Table S3: Means (mg kg^-1^) of LREE, U and Th concentration distributed in the soil profile according to the evaluation times.

| **Depth (cm)** | **2005** | **2008** | **2010** | **2013** | **2005** | **2008** | **2010** | **2013** | **2005** | **2008** | **2010** | **2013** |
| --- | --- | --- | --- | --- | --- | --- | --- | --- | --- | --- | --- | --- |
|  | **-----------------------------mg kg^-1^-------------------------------------** | | | | | | | | | | | |
|  | **La** | | | | **Ce** | | | | **Pr** | | | |
| **0-5** | 71.96bA^1^ | 9.59dC | 37.58cA | 227.69aA | 152.47cA | 141.18cCD | 211.52bB | 480.30aA | 15.32bA | 3.27dC | 9.57cA | 56.57aA |
| **5-10** | 5.83dB | 117.17aA | 25.81cB | 44.28bB | 42.35dB | 304.87aA | 261.41bA | 142.04cB | 1.24dB | 26.63aA | 5.93cB | 12.10bB |
| **10-20** | 3.32cB | 41.10aB | 9.28bC | 9.46bC | 36.77dB | 174.88aB | 151.59bD | 75.00cC | 0.74cB | 9.69aB | 3.03bC | 3.04bC |
| **20-40** | 2.69aB | 4.97aD | 4.38aD | 6.23aC | 37.31dB | 125.01bD | 150.24aD | 73.90cC | 0.62bB | 2.23aC | 2.01abC | 2.37aC |
| **40-60** | 2.23aB | 4.69aD | 4.24aD | 5.27aC | 34.96cB | 156.30aC | 151.69aD | 73.30bC | 0.52bB | 2.20aC | 1.94abC | 2.12aC |
| **60-100** | 2.01aB | 4.80aD | 4.29aD | 4.94aC | 34.27dB | 129.92bD | 149.37aD | 71.25cC | 0.47aB | 2.23aC | 1.96aC | 2.11aC |
| **100-200** | 3.79aB | 5.10aCD | 4.47aD | 5.84aC | 38.91dB | 134.90bD | 171.87aC | 72.54cC | 0.71bB | 2.27aC | 1.98abC | 2.29aC |
|  | **Nd** | | | | **Sm** | | | | **Eu** | | | |
| **0-5** | 56.48bA | 10.57dC | 33.55cA | 214.85aA | 8.35bA | 2.15dC | 5.48cA | 27.38aA | 2.20bA | 1.15cC | 2.03bA | 7.31aA |
| **5-10** | 4.64dB | 95.86aA | 20.78cB | 41.82bB | 0.73dB | 14.42aA | 3.55cB | 6.29bB | 0.13dB | 4.47aA | 1.57cB | 2.12bB |
| **10-20** | 2.75aB | 34.36aB | 10.02bC | 9.50bC | 0.49cB | 5.54aB | 1.96bC | 1.93bC | 0.10cB | 2.08aB | 1.11bC | 1.09bC |
| **20-40** | 2.48bB | 6.94aC | 6.35aC | 7.22aC | 0.40bB | 1.62aC | 1.40aC | 1.67aC | 0.07bB | 1.00aC | 0.98aC | 1.00aC |
| **40-60** | 1.87bB | 6.34aC | 5.88aC | 6.48aC | 0.35bB | 1.58aC | 1.39aC | 1.49aC | 0.06bB | 1.00aC | 0.96aC | 0.96aC |
| **60-100** | 1.99bB | 6.98aC | 6.08aC | 6.32aC | 0.33bB | 1.58aC | 1.41aC | 1.47aC | 0.05bB | 0.99aC | 0.97aC | 0.95aC |
| **100-200** | 2.53bB | 6.99aC | 5.99abC | 7.04aC | 0.40bB | 1.66aC | 1.45aC | 1.58aC | 0.07bB | 1.00aC | 0.98aC | 0.98aC |
|  | **Gd** | | | | **Th** | | | | **U** | | | |
| **0-5** | 8.02bA | 3.69cC | 7.08bA | 24.76aA | 8.94bA | 7.59bA | 8.38bA | 13.58aA | 1.87bA | 2.79aA | 3.11aA | 2.93aA |
| **5-10** | 1.01cB | 14.83aA | 6.61bA | 6.44bB | 8.41aA | 8.46aA | 8.31aA | 9.99aB | 1.75cA | 2.75abA | 3.01aA | 2.44bB |
| **10-20** | 0.78cB | 6.77aB | 3.82bB | 2.92bC | 7.80bA | 7.83abA | 7.96abA | 9.55aB | 1.64cA | 2.78aA | 3.01aA | 2.43bB |
| **20-40** | 0.73bB | 3.16aC | 3.42aB | 2.58aC | 7.67bA | 8.30bA | 8.79abA | 10.23aB | 1.76dA | 2.78bA | 3.16aA | 2.32cB |
| **40-60** | 0.66bB | 3.35aC | 3.46aB | 2.48aC | 7.78bA | 7.81bA | 8.68abA | 9.72aB | 1.69dA | 2.84bA | 3.18aA | 2.22cB |
| **60-100** | 0.64aB | 3.14aC | 3.40aB | 2.46aC | 7.63bA | 8.11abA | 8.83abA | 9.81aB | 1.66dA | 2.81bA | 3.14aA | 2.24cB |
| **100-200** | 0.72bB | 3.24aC | 3.68aB | 2.51aC | 8.36bA | 8.68abA | 9.68abA | 10.30aB | 1.73cA | 3.01aA | 3.32aA | 2.29bB |

1. Means followed by the same lowercase letter in the row and the same uppercase letter in the column do not differ significantly from each other by the Tukey Test at a 5% probability level.

Table S4. Means (mg kg^-1^) of HREE concentration distributed in the soil profile according to four evaluation periods ^(1)^.

|  | **HREE** | | | | | | | | | | | |
| --- | --- | --- | --- | --- | --- | --- | --- | --- | --- | --- | --- | --- |
| **Depth (cm)** | **2005** | **2008** | **2010** | **2013** | **2005** | **2008** | **2010** | **2013** | **2005** | **2008** | **2010** | **2013** |
|  | **mg kg^-1^** | | | | | | | | | | | |
|  | **Sc** | | | | **Y** | | | | **Tb** | | | |
| **0-5** | 19.84aA | 19.33aAB | 21.48aAB | 14.30bB | 8.61bA | 2.66dC | 5.23cA | 14.91aA | 0.69cA | 1.39bcB | 1.64bA | 3.04aA |
| **5-10** | 19.84abA | 17.93bcB | 21.68aAB | 16.35cAB | 1.09cB | 14.62aA | 4.64bA | 4.38bB | 0.06cA | 2.39aA | 1.51bA | 1.61abB |
| **10-20** | 19.16abA | 18.54abAB | 20.88aB | 16.84bAB | 0.78cB | 6.57aB | 2.39bB | 2.04bC | 0.03bA | 1.67aAB | 1.37aA | 1.33aB |
| **20-40** | 20.34abA | 19.24bAB | 22.54aAB | 18.47bA | 0.71bB | 2.15aC | 1.91aB | 1.68aC | 0.03bA | 1.33aB | 1.32aA | 1.31aB |
| **40-60** | 19.85abA | 19.33bAB | 22.40aAB | 17.50bA | 0.61bB | 2.05aC | 1.82aB | 1.47abC | 0.03bA | 1.33aB | 1.32aA | 1.30aB |
| **60-100** | 19.12bA | 19.71abAB | 21.97aAB | 17.40bA | 0.57bB | 2.05aC | 1.82aB | 1.51abC | 0.03bA | 1.33aB | 1.32aA | 1.29aB |
| **100-200** | 20.10bcA | 21.06bA | 24.28aA | 18.14cA | 0.66bB | 2.14aC | 1.88aB | 1.67aC | 0.03bA | 1.34aB | 1.33aA | 1.30aB |
|  | **Dy** | | | | **Ho** | | | | **Er** | | | |
| **0-5** | 2.59cA | 2.49cC | 3.42bA | 7.68aA | 0.35cA | 1.28bB | 1.41bA | 1.87aA | 0.85cA | 2.12bB | 2.40bA | 3.57aA |
| **5-10** | 0.28cB | 6.20aA | 2.92bB | 3.18bB | 0.04cA | 1.79aA | 1.33bA | 1.35bB | 0.10cA | 3.24aA | 2.21bA | 2.29bB |
| **10-20** | 0.18cB | 3.62aB | 2.41bC | 2.30bC | 0.02bA | 1.43aAB | 1.25aA | 1.24aB | 0.07bA | 2.49aAB | 2.05aA | 2.04aB |
| **20-40** | 0.16bB | 2.29aC | 2.24aC | 2.19aC | 0.02bA | 1.25aB | 1.24aA | 1.23aB | 0.08bA | 2.04aB | 2.01aA | 1.99aB |
| **40-60** | 0.15bB | 2.26aC | 2.19aC | 2.14aC | 0.02bA | 1.24aB | 1.23aA | 1.22aB | 0.06bA | 2.03aB | 2.02aA | 1.97aB |
| **60-100** | 0.14bB | 2.29aC | 2.19aC | 2.12aC | 0.01bA | 1.25aB | 1.23aA | 1.21aB | 0.07bA | 2.06aB | 2.02aA | 1.98aB |
| **100-200** | 0.15bB | 2.32aC | 2.23aC | 2.20aC | 0.02bA | 1.24aB | 1.23aA | 1.22aB | 0.08bA | 2.05aB | 2.04aA | 1.98aB |
|  | **Tm** | | | | **Yb** | | | | **Lu** | | | |
| **0-5** | 0.06cA | 2.55bB | 2.57abA | 2.62aA | 0.36cA | 2.37bB | 2.46abA | 2.69aA | 0.039cA | 2.55bB | 2.55bA | 2.58aA |
| **5-10** | 0.006cAB | 2.64aA | 2.56bA | 2.55bB | 0.09cAB | 2.81aA | 2.38bA | 2.39bB | 0.008cAB | 2.59aA | 2.55bA | 2.54bB |
| **10-20** | 0.004bB | 2.58aB | 2.54aA | 2.54aB | 0.08bAB | 2.51aB | 2.34aA | 2.32aB | 0.005bB | 2.56aAB | 2.54aA | 2.54aB |
| **20-40** | 0.006bB | 2.54aB | 2.53aA | 2.53aB | 0.08bAB | 2.37aB | 2.32aA | 2.29aB | 0.007bB | 2.54aB | 2.54aA | 2.53aB |
| **40-60** | 0.003bB | 2.54aB | 2.54aA | 2.53aB | 0.06bB | 2.35aB | 2.32aA | 2.29aB | 0.008bAB | 2.54aB | 2.54aA | 2.53aB |
| **60-100** | 0.003bB | 2.54aB | 2.54aA | 2.53aB | 0.06bB | 2.36aB | 2.31aA | 2.29aB | 0.002bB | 2.54aB | 2.54aA | 2.53aB |
| **100-200** | 0.003bB | 2.54aB | 2.54aA | 2.53aB | 0.07bAB | 2.37aB | 2.31aA | 2.30aB | 0.006bB | 2.54aB | 2.54aA | 2.53aB |

^(1)^ Means followed by the same lowercase letter in the row and the same uppercase letter in the column do not differ significantly from each other by the Tukey Test at a 5% probability level.

1. [↑](#footnote-ref-1)
